# What emerging adults say about the appropriateness of sexual and reproductive health programmes: evidence from a suburb in Accra, Ghana

**DOI:** 10.3389/frph.2024.1459825

**Published:** 2024-12-10

**Authors:** Laud R. Sowah, Adriana A. E. Biney, D. Yaw Atiglo, Delali Badasu, Angela A. Boateng, Kwadwo Ohene Sarfoh, Augustine Ankomah

**Affiliations:** ^1^ Regional Institute for Population Studies, University of Ghana, Legon, Ghana; ^2^ Family Health Division, Ghana Health Service, Accra, Ghana; ^3^ Greater Accra Resilient & Integrated Development (GARID) Project, Accra, Ghana; ^4^ School of Public Health, University of Ghana, Legon, Ghana

**Keywords:** adolescents, emerging adults, reproductive health education, tertiary students, apprentices, informal workers, Ghana

## Abstract

**Introduction:**

Young people's access to appropriate health information in Ghana has been marginal, hence their utilisation of existing services remains poor. Most sexual and reproductive health (SRH) policies and outreach programmes target adolescents, neglecting emerging adults who are equally vulnerable to SRH risks. This study seeks to elicit emerging adults’ knowledge and experiences with SRH programmes, and their recommendations to improve the services for their needs.

**Methods:**

Using data from 30 in-depth interviews and 10 focus group discussions with youth aged 18–24 years in a suburb of Accra, we provide insights on emerging adults’ experiences with SRH programmes and their recommendations for their improvement, as well as young men's perspectives on SRH programmes, in particular.

**Results:**

The participants were in three socio-economic groups: tertiary students, informal workers and apprentices. The main SRH education that the emerging adults had received was from their earlier formal education in Junior and Senior High Schools but not in their current places of school or work. However, they indicate that the SRH education programmes and information they received earlier in life were inadequate to tackle pragmatic issues that contemporary youth face. Furthermore, SRH programmes operate in unfriendly environments with negative messages that cause them to lack vital information.

**Discussion:**

For the success of SRH programmes, the youth should be targeted with diverse contemporary approaches specific for their SRH needs. Key recommendations comprised making available SRH education tailored for emerging adults’ current demographic and socio-economic groups, and providing appropriate SRH content and youth-friendly community centres.

## 1 Introduction

Adolescent sexual and reproductive health and rights (ASRHR) issues such as family planning, induced abortion and sexually transmitted infections are topical challenges which have received global attention and response for the past few decades (1, 2). The 1994 International Conference on Population and Development (ICPD) drew attention to the relevance of acknowledging the urgent and critical sexual and reproductive health (SRH) needs of the youth (1, 2). This has led to significant improvements in ASRH programmes globally over the past three decades. Issues concerning SRH have also been included in the Sustainable Development Goals (SDGs), specifically Goal 3 which seeks to “ensure healthy lives and promote well-being for all at all ages.” SDG targets 3.7 and 5.6 reiterate the need to ensure universal access to quality SRH services (3). By 2030, Target 3.7 seeks to “ensure universal access to sexual and reproductive health-care services, including for family planning, information and education, and the integration of reproductive health into national strategies and programs” while Target 5.6 seeks to “ensure universal access to sexual and reproductive health and reproductive rights” (3).

In Ghana, the policy environment encourages advancements in the health and development of the youth. However, according to the Ghana Adolescent Health Service Policy and Strategy, existing policies on adolescent health have mostly centred on HIV/AIDS prevention and risky sexual behaviour (4). This could be attributed to the high HIV/AIDS prevalence in some parts of sub-Saharan Africa (5) which resulted in a majority of studies focusing on HIV/AIDS prevention and reduction of risky sexual behaviours. This reduces the comprehensiveness of the policy due to its limited ability to explore, prioritise and tackle a wider range of other health issues facing adolescents. An evaluation report on the Adolescent Health and Development (ADHD) programme suggests that adolescents and young people's “access to appropriate health information had not improved significantly over the period of the review [in 2016]”, and the “utilization of health services by adolescents and young people remained poor despite an overall improvement” in some indicators (4).

These policies and programmes, mostly designed as workshops, seminars, educative platforms, and media programmes, are all aimed at addressing the reproductive health challenges of the youth in order to improve their SRH. In Ghana, these programmes are mostly spearheaded by the government through the Ghana Health Service (GHS) with support from international donors, local and international non-governmental organisations, the Ghana Education Service (GES), and religious bodies, to mention but a few. Nonetheless, most of the interventions and policies target early and middle adolescents (6) to the neglect of emerging adults (18–24 years) who are equally vulnerable to sexual and reproductive health risks. The current study does not attempt to undermine the importance of adolescents in the discourse of SRH, but rather to highlight the peculiar SRHR needs of emerging adults. This study has identified the scant or lack of attempt by SRH programme implementers to have deliberate policies or programmes purposefully targeting emerging adults and their SRH needs.

Furthermore, recent literature indicates that very little has been done with respect to male youth and their access to sexual and reproductive health programmes (7–10). Few studies involve boys and men; nevertheless, the need to consider the male perspective is critical as studies indicate that men play a key role in the quest to improve women's health (9–11). Without access to SRH knowledge, young men face challenges of their own, as well as impede partner's contraceptive use, normalise sexual risk-taking behaviour, and succumb to other unhealthy behaviours due to being uninformed (8, 10). These are critical messages in most SRH programmes. For example, some studies indicate that inadequate information or gaps in knowledge, socio-cultural barriers, and insufficient or inapt services for men hindered use of services and weakened support for their partners’ use of SRH services (12–14). Therefore, understanding the male perspective on SRH programmes, their experiences, knowledge and benefits are equally paramount.

Studies conducted with adolescents and youth in various parts of Ghana indicate exposure to *ad hoc* SRH programmes with mixed findings in relation to accurate knowledge on some SRH topics (15–18). Adolescents’ knowledge of different topics ultimately improved after participating in SRH interventions (15, 16, 18). Most adolescents reported challenges with access to SRH services (15, 16, 18). Challenges also existed with parents/caregivers and teachers feeling deficient in their ability to provide SRH education to their wards and students, respectively (15, 17).

Ghana is rapidly urbanising, with the Greater Accra Region being the most urbanised region (19). As urbanisation increases without commensurate equitable health and economic conditions in sub-Saharan Africa (20), the urban youth living in resource-constrained environments will continue to experience limited opportunities and high risks of adverse reproductive health outcomes, including unintended pregnancies (21). The increasing desire to study reproductive health challenges of the youth in such rapidly urbanising settings indicates an enhanced appreciation and understanding of their challenges and developmental stage. Eliciting in-depth and societal level information from young people through qualitative interviews further provides a means of directly generating insights on their viewpoints. Therefore, this study uses in-depth interviews and focus group discussions with emerging adults (18–24 years) to gain insights on their knowledge and/or experiences with sexual and reproductive health programmes, and also seek their views on how current interventions could be improved. This is especially important due to evidence that urban recipients of sexual education are more likely to portray better SRH outcomes (22).

The theoretical perspective from which this study aims to assess the appropriateness of SRH programmes for optimum SRH behaviour is the Health Belief Model (HBM) (23). The HBM is a social cognitive model based on six domains which include perceived threat susceptibility, perceived severity, perceived barriers, perceived benefits, cue to action and perceived self-efficacy (23, 24). The HBM is a behaviour change model that suggests that individuals will take an action to change their behaviour if they believe they can take an action (perceived self-efficacy and cue to action) and, by doing so, will prevent a negative outcome (perceived benefits). In the current study, the perceived threat is represented by negative SRH outcomes. According to the HBM model, the individual, in this context emerging adults, should first understand the negative SRH issues affecting the youth, since the HBM proposes that the emerging adults should be aware of the magnitude of their susceptibility to any poor health outcomes of interest. Previous studies have used the HBM to investigate reproductive health outcomes among young women or adolescents in Ghana but a focus on emerging adults in urban areas including males is largely missing in the literature (25–27). In this study, we utilised the model inductively; and thus, drew from its insights ex post facto, that is, as we interpreted the results.

## 2 Materials and methods

### 2.1 Study design and recruitment

The study used in-depth interview (IDI) and focus group discussion (FGD) data from emerging adults aged 18 to 24 years under an IUSSP Urban Family Planning Project that sought to explore contraceptive use, non-use and discontinuation among emerging adults in the Greater Accra Metropolitan Area. The male and female study participants were categorised into three distinct socio-economic sub-groups: tertiary students, informally employed workers and apprentices. Thirty IDIs comprising 15 males and 15 females were conducted, with five individuals from each socio-economic sub-group. In addition, 10 FGDs were conducted and were segmented by gender and socio-economic stage. The informal worker and apprentice FGDs were also segmented into two age groups 18–20 years and 21–24 years. Interaction is a key component of FGDs; therefore, segmenting the FGDs by age, gender and socio-economic groups, enabled us to group these emerging adults with similar backgrounds/characteristics and behaviours together to better provide opportunities for interaction and discussion within their smaller groupings.

The study was conducted in the La-Nkwantanang Madina Municipal Area (LaNMMA), a municipality in the Greater Accra Metropolitan Area (GAMA), whose capital, Madina, is one of the most densely populated urbanised localities in the municipality. LaNMMA, previously a suburb of the Accra Metropolitan Area (AMA), has grown into a vibrant municipality, housing several young migrants from across the country and neighbouring West African countries (28, 29). Madina has a mix of urban poor and middle-class localities.

Three male and three female interviewers in their mid-to-late-twenties carried out the interviews. All the interviewers had prior experience in conducting IDIs and moderating FGDs. They underwent a five-day training session in November 2020, which provided information about the project as well as information about participant recruitment strategies, administering informed consent, and conducting the IDIs and FGDs. Interview guides were written in English but were practiced in both English and Twi, a popular local language. The IDI guide covered information on the young people's relationship and contraception histories while the FGD guides elicited information on their thoughts about young people's contraceptive use and reproductive behaviour. A pre-test of the study was conducted on the final day of the training, followed by a debriefing session among the study team.

All informal workers and apprentices were selected from various locations in Madina and its environs. They were recruited through several ways for both the IDIs and FGDs. The research team liaised with various trade associations to reach the informally employed and those in apprenticeships. They provided names and contact details of members who were subsequently contacted and informed about the project. Informal workers and apprentices were also approached at their various places of employment in stalls and shops around the busy streets of Madina and in the market. The contact details of those willing to participate were obtained and they were later called and assessed with further criteria for eligibility. For ethical considerations, this was done in order not to single out sexually active participants to engage directly, especially since most recruitment was done while the young people were with their peers. The criteria comprised youth who had ever had sexual intercourse, were between ages 18 and 24 years, and worked in the informal sector or were undergoing an apprenticeship in Madina. Those fitting the inclusion criteria for the study were asked to participate in the study. However, only one of the female apprentices in the older female apprentice FGD who was interviewed was 24-years-old at the time of been recruited but turned 25 by the time of the FGD. This participant was not excluded from the study.

The higher educated students were recruited from two public universities located within four kilometres of Madina. They were recruited through announcements and flyers circulated on the Universities’ email lists and through social media (mainly WhatsApp groups). They were also approached in person on the campuses of the two universities, and contacted through their student unions. The inclusion criteria for tertiary students comprised being sexually active, between ages 18 and 24 years and enrolled in an undergraduate programme at the two universities.

The interviews were conducted between December 2020 and January 2021. The participants deemed eligible to participate were offered the choice of participating in the IDI, FGD or both. For the IDIs, interviewers booked appointments with the interviewees and travelled to meet them in safe but private locations. For the eight informal worker and apprentice FGDs, participants were directed to safe locations in Madina where the FGDs were carried out over a period of four days. The two tertiary student FGDs were carried out at one of the public universities in one day. Interviewers conducted interviews with people of the same sex in the preferred language of the participants which was Twi (a widely spoken local language in Ghana), pidgin English or English. The IDIs lasted between 20 min and 55 min, while the FGDs lasted between about 70 and 135 min. All interviews were audio-recorded, translated and transcribed. The participants were informed about the study and their rights to participate, and they signed consent forms. Upon completion of the interviews all participants were provided with a token of compensation (about $4 worth of phone credit and some refreshments). The researchers obtained ethical clearance for the study from the Ethics Committee for the Humanities at the University of Ghana—ECH135/19-20. See Biney et al. (30) for additional information about the study.

### 2.2 Data analysis

All the interviews were audio recorded and transcribed by the six interviewers. They were then checked for accuracy by a study team member. The study used data analysis software, ATLAS.ti version 7, to carry out the analysis. Three of the research team members employed the use of thematic analysis, to first generate deductive and inductive codes on two transcripts and later build a coding frame. The coding frame guided the coding procedure for the other transcripts. Coding was done separately for the individual and group interviews. Second, patterns were identified within the coded information, and data and codes were grouped into various levels of themes (30, 32). For this paper, the overarching themes of: (1) knowledge and experiences with SRH programmes, (2) recommendations to improve SRH programmes, and (3) young men's perspectives on the topic, had been identified in advance as the three researchers coded the data. One researcher then used the codes generated from the emerging adults’ responses solely on questions asked about SRH programmes to generate the lower-level themes under the three main themes.

## 3 Results

Results from the IDI and FGD participants indicate their knowledge about SRH programmes, sources of their SRH information as adolescents and experiences with these programmes. They also indicated recommendations for improving programmes, while male's perspectives on the programmes were also provided.

### 3.1 Participants’ characteristics

A total of 30 IDI and 72 FGD participants were recruited for the study (see Table 1). Their ages ranged from 18 to 24 years with female IDI participants averaging about one year older than their male counterparts. FGD participants were on the average the same age—21 years. Most of the male informal workers and apprentices had some level of senior high school education while female participants had a mix ranging from no education to some level of senior high school education. Equal numbers (five male and female participants) in each socio-economic group were recruited for the IDIs. Unfortunately, a smaller proportion of male informal workers were recruited for the FGDs than the other groups due to their unavailability, though they initially agreed to participate in the study.

**Table 1:** Characteristics of IDI and FGD participants.

IDI participants	Males	Females
Mean age (age range)	21.5 (19–24)	22.7 (20–24)
Educational attainment		
None	6.7	6.7
Primary	6.7	13.3
Junior High School	6.7	20.0
Senior High School	46.7	26.7
Tertiary	33.3	33.3
Socio-economic groups		
Informally employed	33.3	33.3
Apprentice	33.3	33.3
Tertiary students	33.3	33.3
**Total**	**15**	**15**
FGD participants	Males	Females
Average age (age range)	21.2 (18–24)	21.0 (18–24)
Educational attainment		
None	0.0	5.6
Primary	2.8	8.3
Junior High School	25.0	33.3
Senior High School	36.1	30.6
Tertiary	36.1	22.2
Socio-economic groups		
Informally employed	27.8	38.9
Apprentice	36.1	38.9
Tertiary students	36.1	22.2
**Total**	**36**	**36**

### 3.2 Knowledge and experiences with reproductive health activities and programmes

#### 3.2.1 Programmes, activities and SRH content mentioned and experienced by emerging adults

The findings indicate that the emerging adults received education on SRH from their previous formal education in Junior and Senior High Schools. Participants noted the following:

Yes, I have [attended an SRH programme]…in Senior High School. It was helpful, but I think now you cannot tell someone not to have sex. Rather we should be educated on how to go about it [have safe sex], because I hear stories of my friends, I tell myself if we had been taught all these, they would not have gotten pregnant. So, I have [attended an SRH programme]. (Female tertiary student—IDI)

Like when I was in school, people come to talk to us about reproductive health, and we were also taught in school [by our teachers]. (Young female informal worker—FGD)

The emerging adults mentioned that the talks were centred on preventing sexually transmitted infections/diseases, pregnancy prevention methods through abstinence and contraceptive use, personal hygiene, and sexual abuse.

I learnt you should protect yourself by wearing condoms when having sex to prevent pregnancy and STDs so I learnt a lot. (Female informal worker—IDI)

It [an SRH programme] creates awareness for those who have no knowledge about the effect of not using a contraceptive or things like that. So…it really helped me. (Older male informal worker—FGD)

The participants had knowledge about some programmes and services at the hospitals, most likely the youth-friendly services, but said they rarely used them.

I have heard about hospitals that have counsellors that talk to teenagers about these problems and all that, but I am not… I have never been to them. (Male tertiary student—IDI)

Some had received education from private organisations. One organisation that was mentioned was Marie Stopes International (MSI)—Ghana which is an international non-governmental organisation (NGO) in Ghana that provides reproductive health and family planning education and services across the nation to both men and women. The aim of such organisations is to empower women to choose when they want to have children. This male student was confident that they could support him with information and services, when needed, based on his prior experience with the organisation.

I: What about the Marie Stopes? Why would you go to them?

P: You see they educated me on contraceptives, most at times when they came, all they educated us on is about contraceptives, the use of contraceptives and then condom and stuffs…. I will go to them because I know what they have done before, they have done that before. (Male tertiary student—IDI)

A tertiary student also mentioned a regularly scheduled talk on various youth related topics organised at their university called the “Decoded Show”. In addition, the FGD participants mentioned receiving information from traditional media—television shows with ASRHR messaging, such as the “You Only Live Once (YOLO)”. This is a Ghana Health Service backed TV show for the youth.

#### 3.2.2 Benefits received from some reproductive health activities and programmes

The participants indicated some benefits derived from reproductive health programmes that they had been exposed to, which included topics on how to use a condom, abstinence, and preventing unwanted pregnancy through available family planning services.

So, the benefit I had was if it happens that we are going to have sex we should protect ourselves and one way is using the condom, so with the condom they taught us how to use it or to do family planning and stuff, that helped us. (Male apprentice—IDI)

Some friends in senior high school who had partners accepted and started using the injections and IUD to prevent pregnancy, and after completion I never heard of them getting pregnant. So, it helped them. (Female informal worker—IDI)

I also had a girlfriend who was attending such programmes where they talk about things like that [SRH issues], so when you want to have sex with her, she will tell you, if you do not use a condom, she will not allow you to penetrate. So, through the programme she was aware about pregnancy. (Older male informal worker—FGD)

It was beneficial to me in so many ways. I learnt it was bad for a lady to have an abortion so I decided to abstain at the time. (Male apprentice—IDI)

This latter quote suggests the male apprentice decided to abstain from sex in order to prevent his partners from getting pregnant, with induced abortions becoming a possible outcome of their sexual activity.

#### 3.2.3 There is need for appropriate SRH content and information

Some were of the view that the SRH programmes were tailored in a way that overlooked the views of young people, who are beneficiaries of the programmes. They were therefore, unable to fully appreciate the SRH programmes. For example, one female tertiary student, who dissented from the views shared by other participants, discussed seeking SRH information on her own as programmes available to her were limited to unhelpful information, such as how to wear a sanitary pad. Her response speaks to the risk of mixed messaging that SRH programmes deliver which could harm rather than help the emerging adults.

I think most of them are not open minded, and they are sharing their personal views, while I have an opinion to share, they do not want to listen. They have their view, that is what is supposed to be done so they are not to listening to me …. Most of the things I know today are things I read on my own. All I learnt from these programmes is how well to wear a [sanitary] pad. That is it. (Female tertiary student-IDI)

In such circumstances, when young people find information irrelevant and their questions not being addressed, they may begin to rely on other sources, such as peers or the media for advice, which may be detrimental to their SRH. Subsequent findings further highlight the consequences of this.

Another notable finding was that the SRH information the youth received was sometimes inaccurate and irrelevant for their needs at the time. Female university students tended to report this. One indicated that during her senior high school days, programmes that were organised to address SRH did not tackle relevant and pragmatic issues that contemporary youth are faced with. During these sessions, they were treated as novices; thus, although helpful, the right information was not always given:

We just go back to our rooms and laugh and discuss among ourselves how naïve the teachers think we were. But honestly, some are helpful. (Female tertiary student—IDI)

I remember I went for a programme and we were told that when you have sex your mother will find out. The first day I had sex I was worried and all that because it wasn’t planned but when I got home my mother didn’t notice and I kept looking at her side but she didn’t notice it. That actually made me sexually active because my parents couldn’t notice I had had sex. That was when I realised most of what they said were lies… (Female tertiary student—IDI)

### 3.3 Recommendations to improve SRH education and services for youth

#### 3.3.1 SRH programmes to be made available for university students

One suggestion to improve services for university students was for sexually active individuals to receive frequent education on SRH topics. Some youth had no knowledge on certain SRH topics even as university students.

For example, like me, I didn’t know anything about it [contraception] even though I was in university, level 100, and I didn’t know anything about condoms, emergency contraceptives. Yes, I didn’t know anything about it, I think the education should be more frequent because a lot of youth are out there and they don’t know anything about it. (Male tertiary student—IDI)

The participants also suggested that SRH topics should be included in the university's curriculum since students are unable to abstain. The counselling centre was deemed the appropriate department to teach these topics. One student mentioned how using the curriculum approach would be helpful:

So, I think it should just be added to the curriculum, that’s why the [counselling] centre can have a topic that is meant to educate on this sexual thing. Besides, we are in the university too, we are asked to balance everything, so you balance small [a little] sex, movies and everything is balanced. So, you can’t say you are abstaining. (Female tertiary student—FGD)

#### 3.3.2 Using telehealth services and social media channels

Some university students suggested that demand generation strategies could utilise telehealth services through the use of apps on smart phones, websites, and social media channels. One participant referred to undergraduate students as “online babies” as they preferred such mediums for accessing information. Social media was also mentioned as a mode of informing youth about SRH topics. The need for confidentiality protocols was also emphasised as a requirement through this medium. The young people mentioned that for such programmes to be effective and efficient within the university environment, anonymity and/or confidentiality should be paramount. Even though they spoke of the relevance of having in-person programmes, they believed that in person meetings/programmes would lead to profiling, and negative and judgemental comments from other students, faculty, and programme organisers. Some tertiary students stated:

I don’t know but it [an SRH programme] has its negative effects because some youth will not feel comfortable coming for these programmes. Yeah, but even though it is good, it is informative, they will deliver a lot of information over there but from the youth’s perspective, I don’t think they will come because if they come…[there will be] this kind of impression about [them]… because when I come for these programmes that are maybe sexually related and its effect on the youth, I am coming to learn but somebody somewhere will think that I will come there to learn it and have sex, but it’s to protect myself. (Male tertiary student—IDI)

For the youth, I don’t think they are ready to use the school counselling centre, so there should be a different method or a different way. Yeah, for now, I think they are all on their phones so I think it should be an online educational programme, yeah that one should be okay. (Female tertiary student—IDI)

You know, let me say now we are ‘online babies’ so if there can be something online that the person can just go, easily accessible to share something. This kind of confidentiality being trusted at its peak, I believe if not 100% more will pop up there. (Female tertiary student—IDI)

I believe we all have [university] email addresses…so maybe something like that [SRH information], if it is a paragraph or something, [that is] one method they can use to post it to everyone. (Female tertiary student—FGD]

#### 3.3.3 Master-crafts persons to assist in shaping and training apprentices on their SRH

Apprentices discussed advice received from their master-crafts people (persons that take on and teach apprentices) on SRH issues. An example of advice one female apprentice had received included issues on feminine hygiene, which was deemed important especially for those in relationships.

My madam [master craftsperson] advises me every day, to look neat and presentable as a lady to attract customers and she also told to me to bath twice a day, wash my clothes and I shouldn’t repeat underwear twice when I am in a relationship. And some, because of poverty, do not wear any panties at all. So far as you a lady anytime you get something small (money) save a little in order to purchase your personal items. (Older apprentice females—FGD)

#### 3.3.4 Creation of community friendly centres

The participants felt that the need for information is imperative to the youth. They recommended creating centres from where they can easily access SRH information.

With that, the districts or localities should form groups just like we are discussing today, form groups and meetings which we can attend regularly like twice in a week to educate us more. Because even now you find a twelve-year-old who is pregnant… their mothers do not advise them on these things so people like that do what they want. So, such creation of groups will be very beneficial. (Female informal worker—IDI)

#### 3.3.5 Friendlier and more receptive attitudes should be provided

The participants also advocated for friendlier and more receptive approaches to dealing with the youth during SRH programmes. Parental guidance and advice on SRH were also needed from the beginning, all in a welcoming environment.

I think there should be people willing to talk. Sometimes I just come to campus and I want someone to talk to about my sexual and reproductive health, my mental health, career counselling, but all these Ghanaian people I think are judgmental so I think the youth are scared of talking because they will tag you in a negative way. So, I think people should be open minded, it is not something that happens in a day but we should try not to be judgmental and not judge other people. If I talk to you, you do not have to make me feel like what I am doing is a sin, you need to educate me on it. So, I do it the right way. (Female tertiary student—IDI)

Someone like my madam, when you talk about such things then she calls you names. But when the education is [given] more, people know and appreciate it more. (Female apprentice—IDI)

Our parents, if they should open up with you in the first place, I mean when you are about growing up, they should have a chat with us about it, but then they don’t create that environment so, children often tell their friends or ask for advice from them. Because if you should tell your parent, I think they will kill you. (Female tertiary student—IDI)

These responses suggest that issues of intergenerational conflict regarding SRH are evident in the lives of these young people. Adults are not communicating with their youth about SRH issues as openly as they would like. This leads to a dependence on peers for information and advice.

### 3.4 Male perspectives

#### 3.4.1 Men are left out of the programmes or are viewed as perpetrators

SRH programmes are generally considered critical interventions that have provided immense support to improving young people's health, however, males tend to have varying views. One of such views is that these programmes are mostly strategically done to target young girls while little or no attention is given to the young boys. A male participant mentioned:

They used to say we should avoid the women, the same way the women should avoid us. They mostly call the women and advise them on that. They usually call them to one side to educate them…mostly it was the women they group to one side to advise. (Young male apprentice—FGD)

Others also stated that during such programmes males are made to be seen as the perpetuators or the evil ones who lead their fellow female students astray, and so should be avoided at all cost. The education also included comments that indicated a disapproval of their risky behaviours which they did not like. Despite gaining useful information, the “attacks” on males created perceptions of hostility which deterred them from attending or enjoying the programmes. Young males noted the following:

For us at that time it [SRH education] was like against us. What they were saying was against us, it was against us, so the boys there did not… we were learning one or two of them [SRH information] like the use of condom and others but we were not happy with the rest. (Young male apprentice—FGD)

They were talking about this teenage pregnancy and they should be smart they shouldn’t be following boys who would come and destroy their lives, yeah, that was the main thing they were talking about. They didn’t even say anything about using condom or anything, they just said they [girls] should not follow boys, they were making us look bad…so I said I wouldn’t go for that thing again. (Young male apprentice—FGD)

Okay it was good for me, for this I would say it depends on the kind of person you are, if you are the kind of person who likes to have a lot of sex and all that you would think they are trying to bash you and all that but they are really trying to educate you, you get me? (Male tertiary student, IDI)

These responses from young men suggest a desire to move from perceptions of young women as victims and young men as perpetrators to a gendered viewpoint that emphasises the need to protect both male and female youth in their different ways.

#### 3.4.2 Some young men have no interest in the programmes

In addition, some males mentioned never physically attending any specific SRH programmes, whether at school, religious institutions, associations or through other organised meetings. After denying attending an SRH activity, one male apprentice went on to clarify that his senior high school discussed SRH issues during their orientation.

P: I have not participated in any programme like that before.

I: So you have not attended any programme that they talked about sexual reproductive health before?

P: I think in SHS….it wasn’t really a programme organised for us but it was during the orientation in SHS. So it was during the orientation that the person who was giving us the social talk spoke about that [SRH issues].

I: What did the person say about it? What do you remember was said?

P: It was advice for the youth in the school. How to keep yourself in school, if you are a lady and a guy proposes to you don’t accept because you are a student, etcetera. (Male apprentice—IDI)

Finally, some young men did not see any relevance in such programmes or stated that they were ineffective. They shared their own and their peers’ opinions about SRH programmes:

I: Okay, so what about your friends, what have they said about such programmes. Them attending such programmes what have they said about it?

P: Some of them feel it’s slow, it’s boring…some of them feel it’s very boring.

I: Alright. So, when they say boring, what does that mean?

P: It wastes a lot of time. (Male tertiary student—IDI)

Even my female friends afterwards [after attending the programmes] some of them got pregnant, so with that I don’t see any benefit they got from it. (Male apprentice—IDI)

## 4 Discussion

To the best of our knowledge, this study is the first in Ghana to assess how emerging adults perceive the appropriateness of SRH programmes in Ghana to effectively inform them on the right knowledge and attitudes to engender behavioural change as well as improve access to health care services. Participants expressed an awareness of reproductive health programmes and services. They mostly discussed receiving SRH education during junior and/or senior high school and not at their current places of school or work. Thus, knowledge gained is for those who had the privilege of attending SRH programmes at earlier stages of their life. However, some reported rarely utilising services at the hospital—which they knew were available—with some stating a preference for SRH services offered by private institutions with an SRH mandate. The lack of desire to utilise youth-friendly services available at public health facilities has implications for their reproductive health and meeting global goals. It must be noted that a study using data from sub-Saharan Africa suggests pharmacies and public health facilities as adolescents’ main sources of reproductive health services (33). However, it focused on women's contraception sources and not the range of services provided at facilities, including counselling.

There was a myriad of benefits of SRH activities and programmes to emerging adults. These benefits included protective measures aimed at youth abstaining from sex, appropriate contraceptive use practices, how to wear sanitary pads, etc. That notwithstanding, the content of some health programmes did not reflect the SRH needs of emerging adults. They felt the information provided by their teachers or mentors did not reflect the realities or lived experiences of adolescents at the time, and now as emerging adults. Thus, these young people do not only need SRH information but it must be accurate and age-appropriate. The current content of the programmes also seemed to suggest a target audience of early adolescents, and thus, was not relevant to them. This suggests the content of some programmes should be revised to contain more accurate and useful information for their current needs. Evidence from rural KwaZulu-Natal in South Africa indicates that SRH programmes that provide accurate and appropriate SRH information and services tend to increase the resilience of young people against exposure to HIV and SRH risk (34). Therefore, SRH programmes need to thoughtfully consider providing information suitable for the age and sexual experiences of emerging adults. It is important to also address the challenges males experience with health programmes which tend to be tailored to meet the needs of females (35).

It is for this reason that another key study objective was to interrogate male perspectives on SRH programmes. The findings allude to males being as vulnerable as females based on the views expressed about not accessing services. Young men felt the programmes either side-lined them or accused them of enticing young girls and perpetuating risky sexual behaviour. However, their solution to avoid such programmes puts them at a disadvantage, which may ultimately incur negative consequences for young women. This further highlights the need to move beyond discourses that censure young men to one that targets both male and female youth with solutions to their differing needs (35). The GHS is championing the increase in the demand for family planning by using communication through traditional media to enhance men's participation as well as by engaging male opinion and religious leaders in collaboration with their partners (36). That notwithstanding, the evidence derived from this study suggests more positive information needs to be made available, in this regard, targeting young men.

The study informs us on varied recommendations that youth in the different socio-economic sub-groups proffered. For example, the emerging adults in higher education institutions suggested enhancing the digital approaches for educating the youth as this was the platform most preferred by this group of the youth. The increase of telehealth services targeted at adolescents is evident. Intervention studies especially indicate them as sources of SRH knowledge (37, 38). The GHS has embarked on such interventions (39). For example, the YMK (You Must Know) application has several thousand subscribers and the application features an e-compendium of family planning services and commodities. Providers can refer the youth aptly to provide them access to SRH information (36). However, interventions such as these are targeted primarily to adolescents aged 10–19 years (36). Thus, many emerging adults lack such innovative and age-appropriate interventions to improve their SRH decision-making and behaviour.

The findings from the study reflect various aspects of the HBM. This is simply because SRH programmes are designed to address barriers young people face, expose them to information to take prompt action, present them with benefits they are likely to relish when they engage in choosing right decision, how susceptible they can be, the consequences of taking a negative action and to build their confidence to succeed. This is in line with the six concepts outlined in the HBM. As such, it is evident that SRH programmes adopt some constructs of the HBM theory in its application. With regards to the findings of the study, a major theme like content of messaging relied heavily on the following constructs of the HBM model such as barriers, information to take prompt action, exposure to consequences and benefits and building of confidence. Another theme, that is, knowledge, also made use of “identifying barriers” and “giving information on barriers” constructs of the HBM theory. Finally, a major construct such as confidence building which is one of the key concepts of the HBM theory is expressed in experiences that were shared among the emerging adults in relation to how facilitators of SRH programmes created an atmosphere for them to learn, ask questions and not intimidate them. It was evident again in the experiences they shared, which suggests that if pragmatic issues relating to the lived realities of emerging adults are not discussed, it can affect their confidence in taking the needed choice to overcome a negative consequence.

The emerging adults in this study seemed to be aware of negative health consequences such as HIV/AIDS, unintended pregnancy, and complications from unsafe abortion, to name a few. However, they require age-appropriate and accurate information that emphasises the benefits of avoiding risky behaviour. These should be evident in the messages of the public SRH education provided in their main source of SRH education, secondary schools. This, the findings suggest, may be better received than messages instilling fear and false information. Due to the barriers of inadequate, inappropriate, and hostile messaging, knowledge gained at that level is filled with misinformation, and this affects their current and future attitudes and behaviours. The posture of those providing the information, whether master-crafts persons, parents, teachers, health providers should also be youth-friendly. Despite youth-friendly services being made available at most health facilities, youth do not believe that they truly are accessible to them, due to the attitudes of most adults (4, 40). Therefore, another approach used to educate emerging adults in the informal sector could be peer education. This is a technique used to encourage health-related transformation among peers by sharing health information, principles and actions. Studies have highlighted the importance of peer education in combating HIV/AIDS and risky sexual behaviors in sub-Saharan Africa (41, 42). In addition, Siddiqui et al. (43) suggest that the youth are more probable to believe and initiate advice from their peers. For peer education to be successful in the study setting, it must be supported by two major actors: (i) the Government, more specifically the Ghana Health Service, through the health departments in the La-Nkwantanang Madina Municipality, and (ii) NGOs that deal directly in SRH issues among the youth in Ghana. These group of actors can initiative the anticipated change among emerging adults in the informal sector in Madina.

Stigmatised SRH issues tend to reinforce clandestine sexual activity and response. Further, emerging adults themselves suggested ways to improve SRH education and services with varying suggestions by educational status. The recommendations they made targeted not only the health service supply side but also their parents, peers, mentors and school or work systems. The findings from this study indicate a multi-component and multi-sectoral approach to enhancing the effectiveness of existing SRH programmes for emerging adults in urban areas in Ghana (44, 45). The multi-component approach with regards to generating demand and improved access to SRH services goes beyond the adolescent or emerging adult as an actor to the various roles required to demand generation for the programmes. Essentially, adolescents benefit from different interventions tailored to ensure their demand for the programmes. For instance, university students indicated the benefits of schools using platforms such as orientation sessions, counselling centres, and the curriculum approach—through required university courses—to improve students’ SRH. In addition, the policy implementation structure in Ghana allows collaboration between the GHS and the GES. Such opportunities to impact youth must be further evaluated with appropriate interventions made to improve the services.

Due to limitations of this study, which include the inability to generalise findings to all emerging adults in the three socio-economic groups in the Accra Metropolis, we propose additional research using large scale intervention-based studies targeted at emerging adults to examine appropriate interventions related to SRH messages and mediums to improve their SRH. Despite this limitation, our aim for this study was not to generalise findings but to qualitatively explore these groups of emerging adults’ views on SRH programmes in Ghana.

## 5 Conclusion

Urban emerging adults need SRH information. In order for these programmes to be successful, diverse approaches should be used to provide this information. Also, the communication channels, content of messaging, attitudes of those providing the messages, and platforms used to target the youth must be considered to ensure suitable dissemination of messages to the appropriate audiences. Our study found that barriers to this may result in further promoting sexual activity. The findings further suggest that more avenues are needed for appropriate, accurate and non-judgemental SRH education to be made available by the Ghana Health Service as well as other stakeholders that directly relate with emerging adults across all economic and educational groups (for example, university counsellors and master-crafts persons). Importantly, the adoption and implementation of one policy for youth (10–24 years) must be revised with age-appropriate targets, since some SRH needs differ, as indicated by the findings. In addition, males require tailored and less hostile interventions, while the different categories of youth interviewed (informal workers, tertiary students and apprentices), also require suitable, targeted and evidence-based interventions since evidence indicates their present needs are not being met. Finally, the use of peer education may be an option to address some of the challenges faced by emerging adults as they access SRH programmes.

## Data Availability

The raw data supporting the conclusions of this article will be made available by the authors, without undue reservation.
